# Dynamics of epigenetic regulator gene BCOR mutation and response predictive value for hypomethylating agents in patients with myelodysplastic syndrome

**DOI:** 10.1186/s13148-021-01157-8

**Published:** 2021-08-30

**Authors:** Xiao Li, Feng Xu, Zheng Zhang, Juan Guo, Qi He, Lu-Xi Song, Dong Wu, Li-Yu Zhou, Ji-Ying Su, Chao Xiao, Chun-Kang Chang, Ling-Yun Wu

**Affiliations:** Department of Hematology, Shanghai Jiao Tong University Affiliated Sixth People’s Hospital, Shanghai, 200233 China

**Keywords:** Myelodysplastic syndrome, BCL6 corepressor, Decitabine

## Abstract

**Background:**

BCOR (BCL6 corepressor) is an epigenetic regulator gene involved in the specification of cell differentiation and body structure development. Recurrent somatic BCOR mutations have been identified in myelodysplastic syndrome (MDS). However, the clinical impact of BCOR mutations on MDS prognosis is controversial and the response of hypomethylating agents in MDS with BCOR mutations (BCOR^MUT^) remains unknown.

**Results:**

Among 676 MDS patients, 43 patients (6.4%) harbored BCOR mutations. A higher frequency of BCOR mutations (8.7%) was investigated in patients with normal chromosome, compared to 4.2% in patients with abnormal karyotype (*p* = 0.040). Compared to the BCOR^WT^ patients, the BCOR^MUT^ patients showed a higher ratio of refractory anemia with excess blasts subset (*p* = 0.008). The most common comutations with BCOR genes were ASXL1 (*p* = 0.002), DNMT3A (*p* = 0.114) and TET2 (*p* = 0.148). When the hierarchy of somatic mutations was analyzed, BCOR mutations were below the known initial mutations (ASXL1 or TET2) but were above U2AF1 mutations. Transformation-free survival was significantly shorter in BCOR^MUT^ patients than that in BCOR^WT^ patients (16 vs. 35 months; *p* = 0.035). RNA-sequencing was performed in bone marrow mononuclear cells from BCOR^MUT^ and BCOR^WT^ patients and revealed 2030 upregulated and 772 downregulated genes. Importantly, HOXA6, HOXB7, and HOXB9 were significantly over-expressed in BCOR^MUT^ patients, compared to BCOR^WT^ patients. Eight of 14 BCOR^MUT^ patients (57.1%) achieved complete remission (CR) with decitabine treatment, which was much higher than that in BCOR^WT^ patients (28.7%, *p* = 0.036). Paired sequencing results (before and after decitabine) showed three of 6 CR patients lost the mutated BCOR. The median survival of CR patients with a BCOR^MUT^ was 40 months, which was significantly longer than that in patients with BCOR^WT^ (20 months, *p* = 0.036). Notably, prolonged survival was observed in three BCOR^MUT^ CR patients even without any subsequent therapies.

**Conclusions:**

BCOR mutations occur more frequently in CN MDS patients, predicting higher risk of leukemia transformation. BCOR^MUT^ patients showed a better response to decitabine and achieved longer post-CR survival.

**Supplementary Information:**

The online version contains supplementary material available at 10.1186/s13148-021-01157-8.

## Background

The myelodysplastic syndrome (MDS) is an epigenetic disease characterized by increased stem cell proliferation coupled with aberrant differentiation resulting in a high rate of apoptosis and eventual symptoms related to bone marrow failure [1]. Cellular differentiation is an epigenetic process that requires specific and highly ordered DNA methylation and histone modification programs. Aberrant differentiation in MDS can often be traced to abnormal DNA methylation as well as mutations in genes that regulate epigenetic programs involved in DNA methylation or histone modification control [1].

The BCL6 corepressor (BCOR) is located on chromosome X, in the Xp11.4 locus, taking part in a specific type of polycomb repressive complex (PRC) 1.1 that mediates transcriptional repression through epigenetic modifications of histones. BCOR also functions as an interacting corepressor of BCL-6 that enhances BCL-6-mediated transcriptional repression. Therefore, constitutional inactivating mutations of this gene have been implicated in many solid tumors and hematopoietic malignancies, including leukemia and MDS (perturbing myeloid differentiation and promoting leukemogenesis) [2, 3].

The diversity of somatically mutated genes reflects a variety of pathogenic mechanisms in MDS [4–7]. Among the various somatic mutations during the development of MDS, BCOR mutations were reported in detail until 2013 [8] and may carry some prognostic value [8–10]. BCOR mutations appeared to occur easily in MDS patients with normal chromosomes; BCOR mutations were associated with DNMT3A or RUNX1 mutations and were independently associated with worse overall survival (OS). However, there are few reported cases, and the response status of BCOR^MUT^ patients towards classical hypomethylating agents (HMAs) is still lacking.

In this assay, the similarities and differences of the BCOR mutation features of a large cohort (676 total patients) of Chinese MDS patients were analyzed and compared to the results in the existing literature. More importantly, we analyzed for the first time the response of a group of 14 BCOR^MUT^ patients to the standard decitabine treatment. Furthermore, the possible mechanism of BCOR mutation in the pathogenesis of MDS was explored primarily.

## Methods

### Subjects

A cohort of 676 patients was included continuously in this study. All the patients were diagnosed in our own center between January 2009 and March 2019. According to the World Health Organization (WHO) and French–American–British classification (FAB) [11], this cohort included 646 MDS patients and 30 chronic myelomonocytic leukemia (CMML) patients. Clinical and hematologic data were recorded following informed consent in accordance with the Declaration of Helsinki; this study was approved by hospital review boards of the Shanghai Jiao Tong University Affiliated Sixth People’s Hospital.

### Targeted gene sequencing

Genomic DNA (gDNA) was extracted from bone marrow mononuclear cells. The purity (OD260/280 > 1.8) and concentration (50 ng per µl) of the gDNA met the sequencing requirements. We used a gene panel to amplify 39 MDS-related target genes: ANKRD11, ASXL1, BCOR, CBL, CEBPA, CALR, DHX9, DNMT3A, ETV6, EZH2, FLT3, GATA2, IDH1, IDH2, ITIH3, JAK2, KIF20B, c-KIT, KRAS, MPL, NF1, NPM1, N-RAS, PHF6, PTPN11, PTPRD, ROBO1, ROBO2, RUNX1, SETBP1, SF3B1, RSF2, STAG2, TET2, TP53, U2AF1, UPF3A, WT1 and ZRSR2. These targeted genes were sequenced for mutations using MiSeq sequencing (Illumina, San Diego, CA, USA). To identify mutations in the highlighted genes, we designed PCR primers using the primer XL pipeline. A total of 1164 oligonucleotide pairs were produced. The amplification reactions were conducted using an ABI 2720 Thermal Cycler. The PCR products were used to generate a library for further detection, and the DNA adapter-ligated and adapter-indexed fragments from 10 libraries were then pooled and hybridized. After hybridization of the sequencing primers, base incorporation was performed in a single lane using a MiSeq Benchtop Sequencer following the manufacturer's standard cluster generation and sequencing protocols; in total, 250 cycles of sequencing per read were used to generate paired-end reads, and included 250 bp at each end and 8 bp of the index tag.

### RNA sequencing and analysis of integrative network

Total RNA was extracted with TRIzol reagent (Invitrogen, CA, USA) following the vendor's process from bone marrow mononuclear cells of three MDS patients harboring BCOR mutations and three patients with BCOR wild type. RNA sequencing was performed on the Illumina Hiseq 2500 (Illumina, USA). R software package was adopted for processing the differentially expressed genes (DEGs). Pathway enrichment analyses of the dysregulated genes were also carried out. Our study employed the Gene Ontology (GO) and Kyoto Encyclopedia of Genes and Genomes (KEGG) online database to identify the interactions of DEGs.

### Decitabine therapy

A total of 195 of the 676 MDS patients accepted decitabine treatment (DACOGEN; JANSSEN PHARMACEUTICA), and their responses and outcomes were retrospectively analyzed. Enrolment (beginning of decitabine therapy for each individual) occurred between September 2009 and January 2019. All 195 patients received 20 mg/m^2^ decitabine intravenously for 5 consecutive days every 4–6 weeks. The median therapy course was four cycles. Among these patients, fourteen harbored BCOR mutations. The median number of decitabine therapy cycles for the 14 BCOR^MUT^ patients was also four. Response to treatment was assessed using the International Working Group (IWG) Response Criteria for MDS, which was revised in 2006 [12].

### Statistical analysis

Statistical analyses were conducted using SPSS software, version 19.0. The association of mutations with clinical characteristics was analyzed via the *χ*^2^ test. Comparisons of two independent samples were assessed using two-tailed Student’s *t* test. OS was defined as the time from the date of diagnosis to death or survival at the last follow-up (censored). Acute myeloid leukemia (AML)-free survival was calculated from the time of diagnosis to AML progression at the last follow-up (censored). Cumulative incidence of AML transformation was documented. Kaplan–Meier analysis was used to evaluate the time of survival and time of transformation to AML. All *p* values were based on 2-sided tests, and *p* values less than 0.05 were considered statistically significant.

## Results

### Mutation landscape of BCOR

**Fig. 1:**
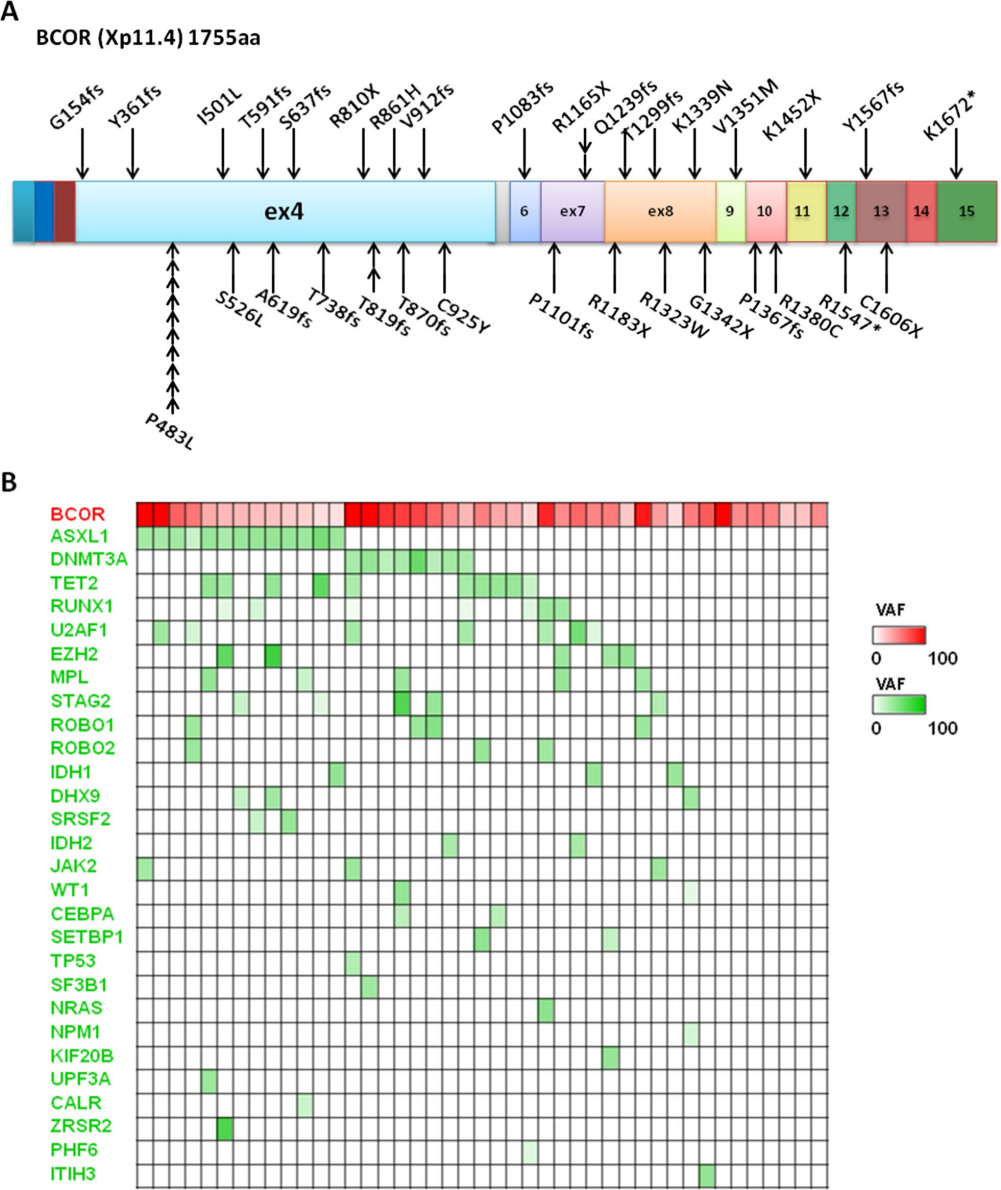
BCOR mutations in 43 MDS patients. **a** Localization of mutations identified in the BCOR gene. Each mutation is shown with an arrow. Exons of the BCOR gene are shown in colored boxes as indicated. **b** Co-occurrence of BCOR mutations with other gene mutations studied in 43 MDS patients. The intensity of the color indicates VAF of the gene mutations

**Fig. 2:**
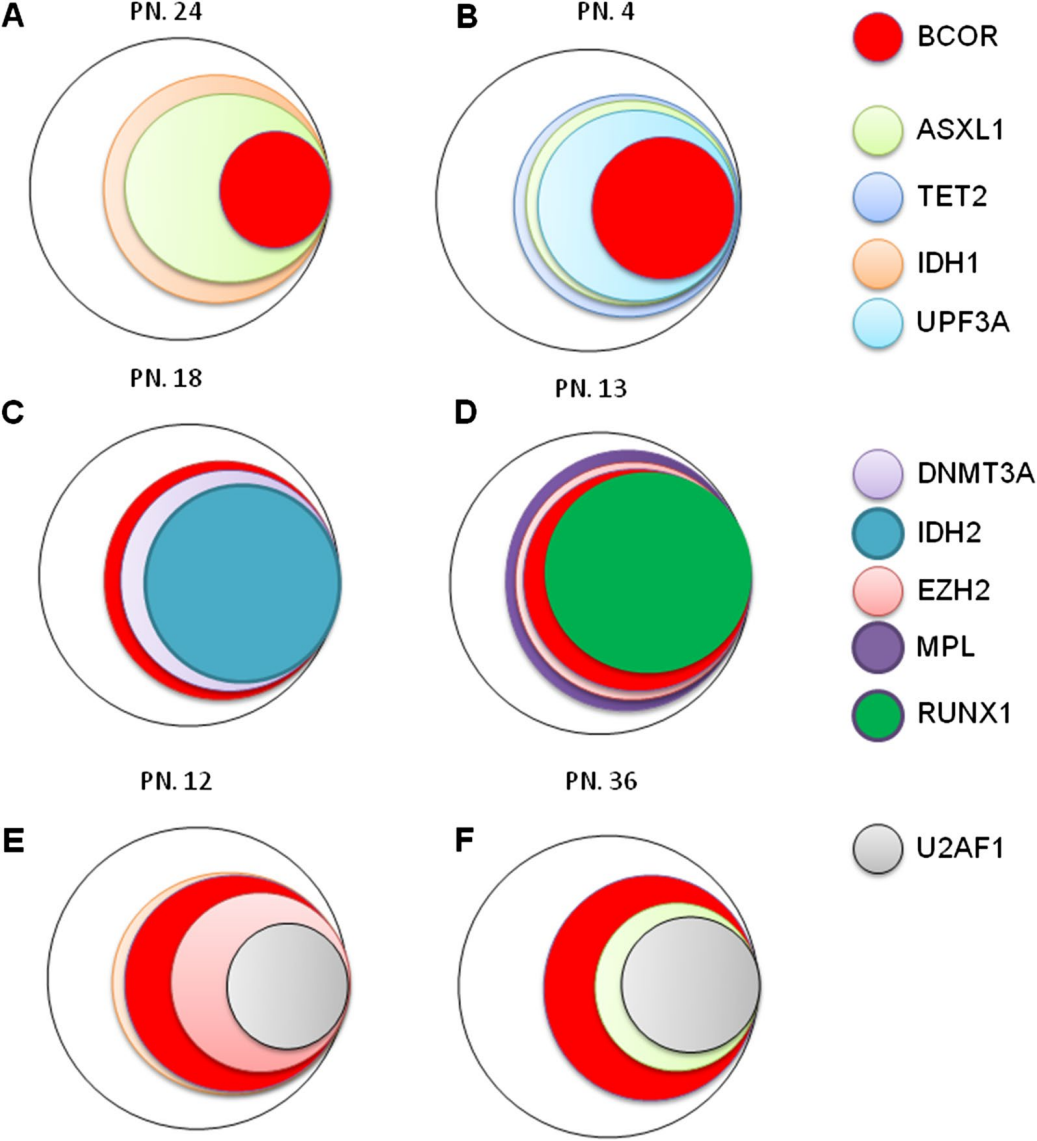
Repartitioning of BCOR mutations and other mutations. For each gene, the percentage represents the estimated percentage of cells carrying the unique mutation. Panels **a** and **b** show examples of lower VAFs for BCOR mutations than other co-mutated genes (such as ASXL1 or TET2) in 2 patients. Panels **c** and D show BCOR mutations that are parallel with other mutations (DNMT3A or IDH2 mutations). In panels **e** and **f**, BCOR mutations presented as earlier events than other mutations (such as U2AF1 mutations). *PN* presented patient number in Additional file 1: Table S1

Among the 676 patients, a total of 43 patients (6.4%) displayed BCOR gene mutations. Twenty-five of the 43 (58.1%) mutations involved exon 4, and ten of the 25 patients harbored a P483L mutation. Nineteen (44.2%) of the 43 mutations gave rise to a truncated BCOR gene [including 16 patients (37.2%) with a frameshift mutation] (Fig. 1a). Six of the 43 patients presented isolated BCOR mutations, and all the remaining BCOR mutations coexisted with mutations in other genes, most significantly including ASXL1 (13/43 BCOR^MUT^ patients, 30.2%, vs. 84/633 BCOR^WT^ patients, 13.3%; *p* = 0.002), DNMT3A (8/43 BCOR^MUT^ patients, 18.6%, vs. 68/633 BCOR^WT^ patients, 10.7%; *p* = 0.114), and TET2 (10/43 BCOR^MUT^ patients, 23.3%, vs. 95/633 BCOR^WT^ patients, 15.0%; *p* = 0.148). Mutation of RUNX1 (reported by Frederik Damm [7] to be the most common comutation was inconspicuous in this assay (7/43 BCOR^MUT^ patients, 16.3%, vs. 95/633 BCOR^WT^ patients, 15.0%; *p* = 0.822) (Fig. 1b). To obtain insights into the hierarchy of somatic mutations in BCOR^MUT^ patients, we investigated all identified comutations in bulky BM cells from 14 female BCOR^MUT^. Patients with isolated BCOR mutations were not included because of the lack of co-mutated genes. Male patients were also ruled out from this analysis for their mutated BCOR variant allele frequency (VAF) appeared to be bi-allele-alike, because of the special location of BCOR in chromosome X. BCOR mutations emerged in three styles (Fig. 2): First, the VAF of BCOR mutations was lower than that of the coexisting mutations, suggesting that the BCOR mutations were not disease-initiating events (especially comutated with ASXL1 and TET2) (Fig. 2a, b). Second, BCOR mutations were parallel with other mutations (Fig. 2c, d). Third, BCOR mutations presented as earlier events than some of the other mutations (such as in U2AF1) (Fig. 2e, f).

### Clinical phenotypes of BCOR^MUT^ patients

Compared to BCOR^WT^ patients, the 43 BCOR^MUT^ patients showed the following features: much higher recurring frequency in patients with normal chromosome (NC) (31/43 BCOR^MUT^ patients vs. 355/633 BCOR^WT^ patients, *p* = 0.040); higher refractory anemia with excess blasts (RAEB) subset ratio (*p* = 0.008); and tending to have more ≥ 1.5 of international prognostic scoring system (IPSS) scoring (*p* = 0.093) (Table 1).

**Table 1:** Comparison of clinical parameters between BCOR^MUT^ and BCOR^WT^ patients

Characteristics	BCOR mutated (*n* = 43)	BCOR wild-type (*n* = 633)	*p*
Age (mean) (year)	55.8	55.9	0.970
Sex (M/F) (no) (ratio)	24/19 (1.3)	371/262 (1.4)	0.719
Normal chromosomes (*n*) (%)	31/43 (72.1)	355/633 (56.1)	*0.040*
RAEB (1 + 2) (*n*) (%)	23/43 (53.5)	174/633 (27.5)	*0.008*
IPSS ≥ 1.5 (*n*) (%)	15/43 (34.9)	149/633 (23.5)	0.093
AML transformation ratio (*n*) (%)	11/43 (25.6)	97/633 (15.3)	0.076
Median overall survival (*m*)	32	36	0.214
AML free survival (*m*)	16	35	*0.035*

The *p* value less than 0.05 is expressed in italics, suggesting a statistical difference

### Prognostic impact of BCOR mutations

**Fig. 3:**
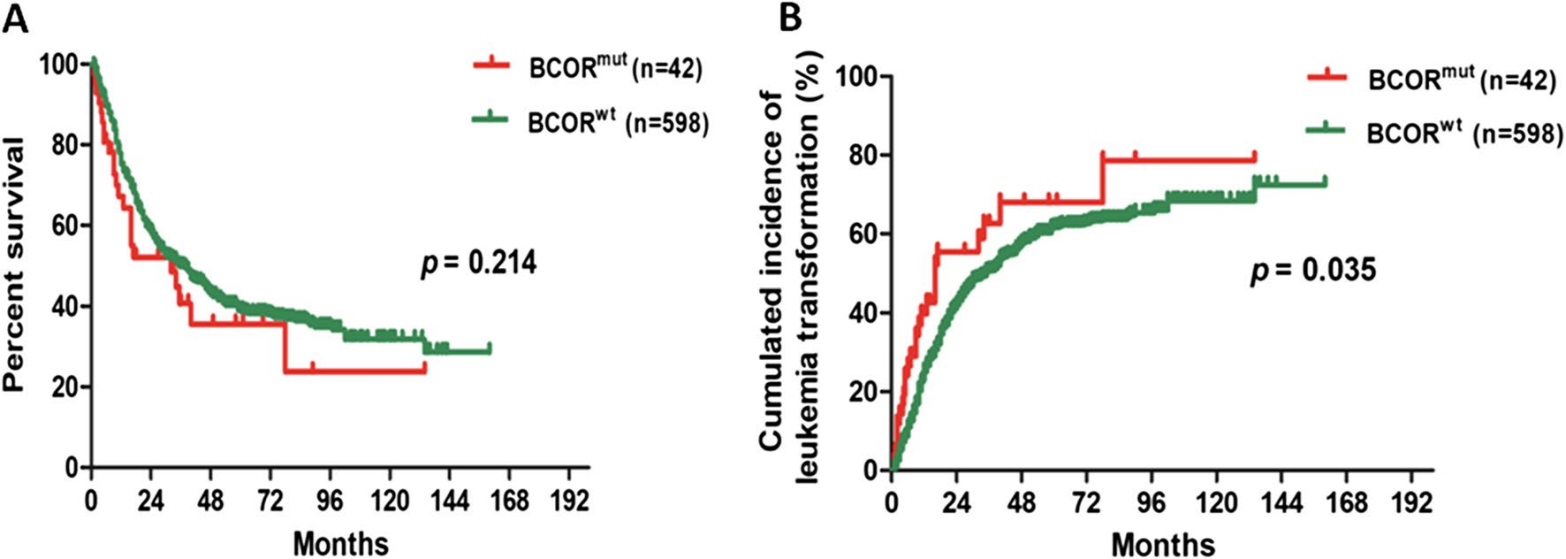
The overall survival and the cumulative incidence of AML transformation in patients harboring BCOR mutations versus patients without BCOR mutations. **a** Kaplan–Meier curve for OS and **b** cumulative incidence of AML transformation according to BCOR mutation status

The prognostic impact of BCOR mutations was evaluated. The median follow-up duration was 21.5 months. Although insignificant, in univariate analysis, a higher cumulative AML transformation rate occurred in BCOR^MUT^ patients (25.6% of the BCOR^MUT^ patients vs. 15.3% of the BCOR^WT^ patients; *p* = 0.076) (Table 1), and a relatively shorter OS was also observed in this BCOR^MUT^ subset (32 months for BCOR^MUT^ patients vs. 36 months for BCOR^WT^ patients;* p* = 0.214) (Fig. 3a). Notably, transformation-free survival (TFS) was significantly shorter in BCOR^MUT^ patients than in BCOR^WT^ patients (16 vs. 35 months; *p* = 0.035) (Table 1 and Fig. 3b).

### Dynamics of BCOR and concurrence gene mutations during AML transformation

Serial BM targeted gene sequencing was performed in six patients with MDS harboring BCOR mutations before and after AML transformation. The molecular changes involved in the transformation of MDS to AML showed two patterns of clonal evolution. The first pattern was obtaining new gene mutations (transformation-related mutations) based on BCOR mutation, involving tumor signaling molecules, transcription factors or tumor suppressor genes. The second pattern was that the VAF of existing transformation-related gene mutations increased with time. Four of six patients showed as the first pattern. (Additional file 1: Fig. S1A–D. The VAF of BCOR mutation and other concurrence mutated genes were showed in red and green bar, respectively. The black bar showed the new gene mutations obtained in the process of AML transformation). Two of six patients presented as the second pattern of clonal evolution (Additional file 1: Fig. S1E and F. The black bar showed existing transformation-related mutations increased with time).

### BCOR mutation affects gene expression profiles of marrow cells

**Fig. 4:**
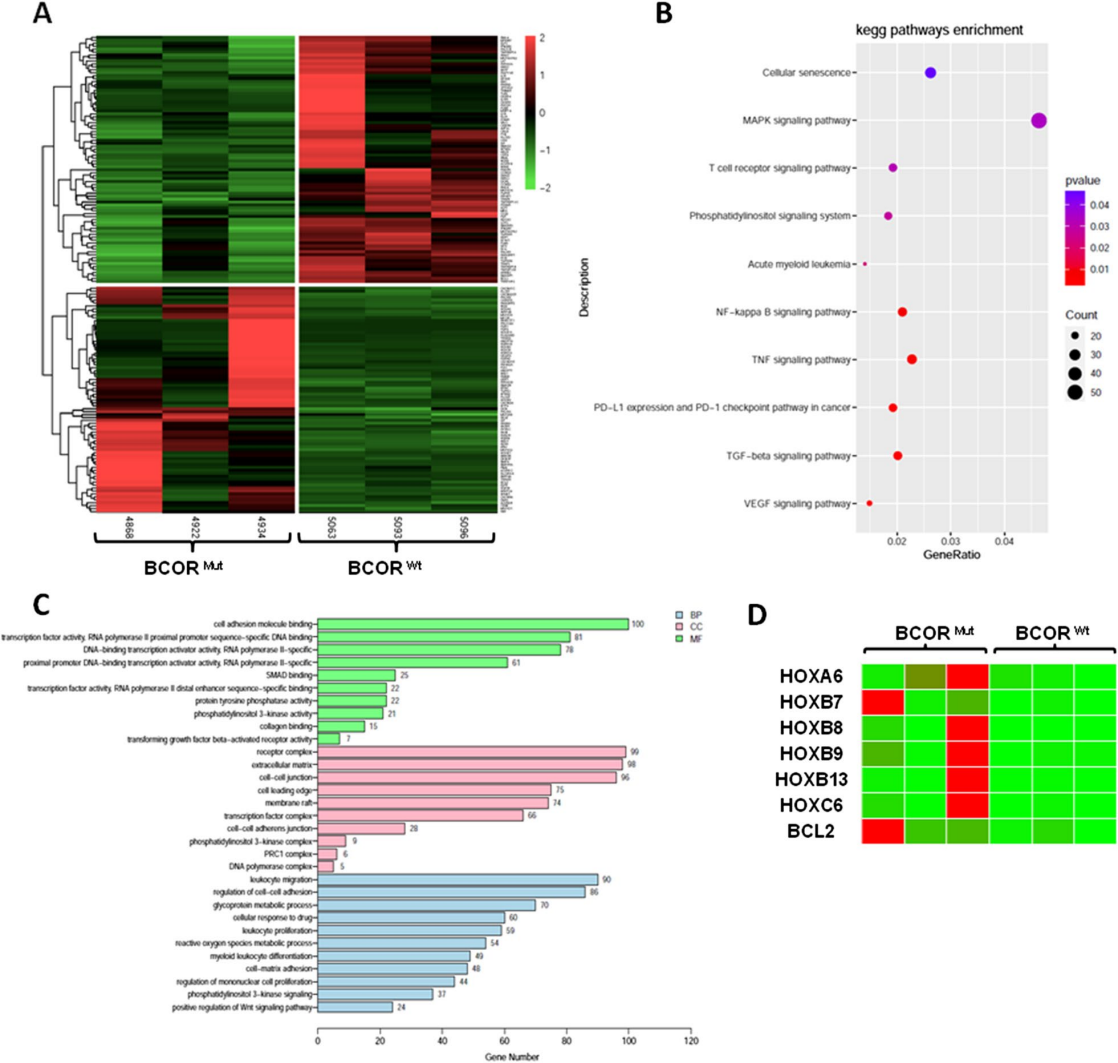
RNA-sequencing analysis in patients with BCOR^MUT^ and BCOR^WT^ **a** Hierarchical clustering plot of the fold changes of gene expressions in three MDS patient with BCOR^MUT^ and three cases with BCOR^WT^ detected by RNA-sequencing (FC > 1.5, *p* values < 0.05). **b** KEGG pathway enrichment analysis **c** The top 10 enriched GO terms in biological process, cellular component, and molecular function. **d** HOX cluster genes including HOXA6, HOXB7and HOXB9 were significantly over-expressed in patients with BCOR^MUT^

To understand how the BCOR mutation promotes MDS, we next compared independent RNA samples of bone marrow mononuclear cells (MNC) from patients with BCOR mutation and wild type using RNA-sequencing (RNA-seq). RNA-seq data revealed 2802 differentially expressed genes (DEGs), including 2030 upregulated and 772 downregulated genes (fold changes (FC) cut-off of 35 1.5; *p* < 0.05) (see Additional file 2). Through RNA-seq of three pairs of independent RNA samples from bone marrow MNC, we determined the transcriptome landscape and identified the potential core genes based on hierarchical clustering analysis. The results of hierarchical clustering (Fig. 4a) suggested that the mRNA expression patterns were distinguishable between the BCOR^MUT^ and BCOR^WT^ cases. GO analyses covered three domains: biological process, cellular component and molecular function. The top 10 enriched GO terms in biological process, cellular component, and molecular function were shown in Fig. 4c. The results of KEGG pathway analysis are presented in Fig. 4b. The genes of differentially expressed were mainly associated with NF-KB pathway and MAPK signal pathway, etc. Importantly, HOX group genes including HOXA6, HOXB7and HOXB9 were significantly over-expressed in patients harboring BCOR mutation, compared to BCOR wild type patients (Fig. 4d).

### Treatment response of BCOR^MUT^ patients to decitabine

**Fig. 5:**
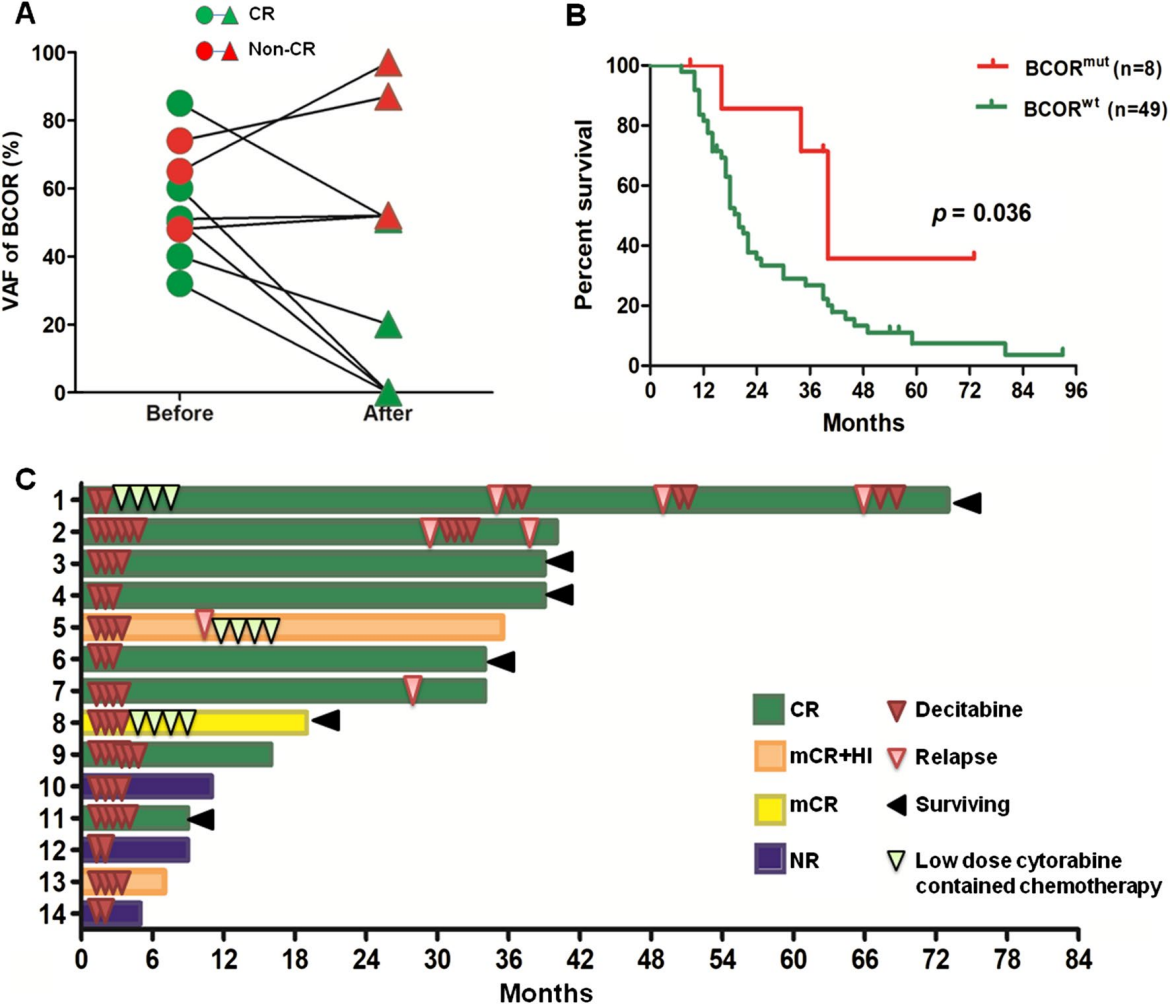
Responses, mutation burden changes and survival status of patients harboring BCOR mutations treated with decitabine. Panel **a** shows the VAF changes from before to after decitabine therapy. Green circles indicated for CR patients and red circles for non-CR patients. Panel **b** shows the overall survival of BCOR^MUT^ and BCOR^WT^ patients who received decitabine therapy. In Panel **c**, the decitabine administration, response and survival status are indicated

A total of 14 BCOR^MUT^ patients accepted the decitabine treatment protocol. The baseline characteristics did not differ substantially between patients with BCOR^MUT^ and BCOR^WT^ (Table 2). The median cycles of decitabine therapy between the two groups was both four (*p* = 0.411, Table 2). Eight of the 14 patients achieved complete remission (CR) (57.1%), which was much higher than that in patients without BCOR mutations (52 of 181 patients, 28.7%) (*p* = 0.036). Three BCOR^MUT^ patients achieved marrow CR (mCR) or mCR with hematological improvement (HI). The remaining 3 patients showed no response to decitabine (Table 3). When paired sequencing results (before and after decitabine) (six sets of sequencing results were available from the 8 patients who achieved CR) were analyzed, three patients showed a loss of the mutated BCOR, two showed a decrease in the VAF of mutated BCOR, and only one showed an unchanged BCOR mutation. In contrast, the three patients without a CR (non-CR) for whom paired sequencing results were available had an unchanged or increased VAF of mutated BCOR (Fig. 5a).

**Table 2:** Characteristics of patients with BCOR^MUT^ or BCOR^WT^ received decitabine therapy

Characteristics	BCOR^WT^ (*n* = 181)	BCOR^MUT^ (*n* = 14)	*p* value
Age, median (range), year	62 (17–85)	62 (23–80)	0.558
Gender, male:female, *n* (%)	120:61 (66:34)	7:7 (50:50)	0.250
WHO/FAB classification, *n* (%)			0.891
RCMD	47 (26.0)	3 (21.4)	
RAEB-1	57 (31.5)	3 (21.4)	
RAEB-2	39 (21.5)	5 (35.7)	
RAEB-t	12 (6.6)	1 (7.1)	
CMML-1	17 (9.4)	1 (7.1)	
CMML-2	9 (5.0)	1 (7.1)	
IPSS risk, *n* (%)			0.858
Intermediate-1	84 (46.4)	7 (50.0)	
Intermediate-2	77 (42.5)	5 (35.7)	
High	20 (11.1)	2 (14.3)	
Cytogenetic risk group, *n* (%)			
Good	108 (59.7)	12 (85.7)	0.072
Intermediate	31 (17.1)	2 (14.3)	
Poor	42 (23.2)	0	
Decitabine cycles, median (range), *n*	4 (1–11)	4 (2–7)	0.411

**Table 3:** Gene mutation features of 14 BCOR^MUT^ patients who accepted decitabine induction

Case no	Class	Cycles*	Response	BCOR mutations (VAF)	Concomitant mutations
1	RAEB-t	2	CR	BCOR/4/C925Y (51%)	DHX9; WT1
2	RAEB1	6	CR	BCOR/11/K1452X (60%)	DNMT3A; ROBO1; STAG2
3	RAEB2	4	CR	BCOR/8/R1323W (50%)	NPM1
4	CMML1	3	CR	BCOR/8/K1339N (33%)	ASXL1; MPL; TET2; UPF3A
5	RAEB1	4	mCR + HI	BCOR/4/A619fs (16%)	PHF6; RUNX1; TET2
6	RCMD	3	CR	BCOR/4/Y361fs (85%)	NRAS; ROBO1; RUNX1; U2AF1
7	RCMD	4	CR	BCOR/12/R1547*(49%)	ASXL1; RUNX1; SRSF2
8	RAEB1	4	mCR	BCOR/4/p819-819del (74%)	DNMT3A; IDH2; ROBO1; RUNX1
9	RAEB2	4	CR	BCOR/7/R1165X (29%)/11/C4326 + 1C > A (56%)	CEBPA; DNMT3A; RUNX1; STAG2;TET2; U2AF1
10	RAEB2	4	NR	BCOR/7/P1101fs (65%)	ITIH3
11	RAEB2	4	CR	BCOR/4/V912fs (32%)	ASXL1; STAG2
12	RCMD	2	NR	BCOR/4/P483L (48%)	DHX9; EZH2; IDH1; U2AF1
13	RAEB2	4	mCR + HI	BCOR/4/T738fs (46%)	EZH2; MPL; RUNX1
14	CMML2	2	NR	BCOR/4/P483L (100%)	None

^*^The induced decitabine cycles number means the course number of continuously accepted decitabine treatment (the durations between courses did not exceed 30 days); For the CR achieved patients with less than 4 induced courses. (No. 1, 4 and 6), the patients rejected continuously decitabine usage by themselves

We hope to find a special mutated locus for BCOR or some special concomitant mutations that may play roles in good or poor responses to decitabine for these 14 patients. Table 3 shows that patients with the mutation of P483L in BCOR on exon 4 (No. 12 and 14) did not have a response to decitabine. Among the 8 patients who achieve CR, three showed coexisting ASXL1 mutations (No. 4, 7 and 11) (Table 3).

In addition to a higher response, the median survival of CR patients with a BCOR^MUT^ was 40 months, which was significantly longer than that in patients without a BCOR mutation (20 months, *p* = 0.036) (Fig. 5b). Notably, prolonged survival (not accompanied with any relapse signs) was observed in three BCOR^MUT^ CR patients even without any subsequent therapies after receiving 3–5 cycles of decitabine (Nos. 3, 4 and 6 in Fig. 5c). The other CR patient, who accepted only 2 courses of decitabine or dose-reduced cytarabine-containing chemotherapy when the disease showed early relapse, has survived for 73 months (No. 1 in Fig. 5c).

## Discussion

In our assay, BCOR mutations occurred in 6.4% of MDS patients (including CMML) (forty-three out of 676 patients), which is similar to the data reported by Frederik Damm et al. [8] (4.7% mutation rate in 408 MDS/CMML patients). Similarly, our assay identified that 44.2% of patients had truncating mutations (including 37.2% with frameshift mutations and 7% with other inactivating mutations), supporting the theory that BCOR mutations can be an independent pathogenic factor [8]. In addition, as referred to by several previous reports [13–15], our results showed, among the 43 patients with BCOR mutations, 71.2% of the individuals possessed normal chromosomes (*p* = 0.040 when compared to those without BCOR mutations). Interestingly, we found that BCOR mutations were frequently associated with mutations in ASXL1/DNMT3A and TET2 but not in RUNX1 (*p* = 0.822) (Fig. 1b); the *p* value for the association between BCOR and RUNX1 mutations was 0.002 as reported by Frederik Damm et al. [8], which was not similar to that for ASXL1/DNMT3A and TET2, which play initial roles in the origins of MDS [16] and also not similar to U2AF1/RUNX1 and TP53, which are linked with unique abnormal chromosomes [17–19] (trisomy 8; chromosome 7 involvement; and complex karyotypes). BCOR mutations appear to be located between the MDS origin-related mutations and MDS phenotype-/transformation-related mutations (Fig. 2), as can be seen from our hierarchical analysis of somatic mutations. For the most part, BCOR mutations occurred as later events following ASXL1 or TET2 mutation (Fig. 2a, b). In another condition, BCOR mutations appeared as an earlier event, especially when comutated with U2AF1 (Fig. 2e, f). Occasionally, BCOR mutations coemerged with other mutations (such as DNMT3A or IDH2) as co-origins of MDS (Fig. 2c, d). Deeper functional studies are necessary to explore the role that BCOR mutation plays during MDS development.

RNA-sequencing results showed that HOX family genes including HOXA6, HOXB7 and HOXB9 were significantly over-expressed in patients with BCOR^MUT^, compared to BCOR^WT^ patients. BCOR takes part into one specific type of polycomb repressive complex PRC1.1 that mediates transcriptional repression through epigenetic modifications of histones by adding an ubiquitin to histone H2A at lysine 119. This process (among others) leads to a silencing of HOX gene clusters [20] and mediates transcriptional repression. Loss of function mutation in BCOR results in the transcriptional activation of targeted genes. In the current study, HOXA6, HOXB7 and HOXB9 increased in patients with BCOR^MUT^, which indicated HOXA6, HOXB7 and HOXB9 maybe the targeted molecular of BCOR mutation in MDS. Subsequent studies will be needed to confirm HOX genes as target of BCOR mutations mediated in leukemia transformation of MDS.

According to the literature, BCOR mutations usually predict poor prognosis [9, 10]. In our assay, 43 BCOR^MUT^ patients presented poor clinical features as a higher ratio of patients with higher IPSS scores (≥ 1.5) (*p* = 0.093) and a higher risk of MDS in the RAEB subset (*p* = 0.001) (Table 1). Though insignificantly, BCOR^MUT^ patients showed a higher ratio of AML transformation and a shorter OS time than those with BCOR^WT^ (see Table 1). Importantly, BCOR^MUT^ patients presented significantly shorter AML-free survival (16 vs. 35 months; *p* = 0.035). The high frequency of the RAEB subset of BCOR^MUT^ patients may cause more AML transformation as well as shorter TFS and OS. The reason for the insignificant difference of OS when compared to controls may come from the good response of some BCOR^MUT^ patients to decitabine therapy as described below.

The dynamic analysis of gene mutations in patients who transformed to AML showed that there were two patterns of clonal evolution, one was obtaining new gene mutation based on BCOR mutations, and the other was that the VAF of other genes that concurrence with BCOR mutations increased significantly. It hinted that BCOR mutations itself may not lead to direct leukemic transformation of clonal cells, but on the basis of BCOR mutation, clonal cells have a disposition to obtain other leukemic transformation related gene mutations, or lead to further accumulation of other leukemic transformation genes, which lead to drive MDS to transform to AML.

It is well known that the response duration achieved for MDS patients by decitabine was only approximately 6–24 months despite regular subsequent treatments, resulting in only a median OS of 18 months [21, 22]. And Patients who do not respond to HMAs, or those in whom the disease progresses after an initial response, have a poor outcome [23, 24]. Under this premise, HMAs plus other drugs [25–27] (with different mechanisms) may be one of the ways to achieve a longer survival rate. In addition, looking for some gene mutations showing special sensitivities to HMAs could be regarded as one of the ways to achieve a better prognosis. In 2017, we reported, although with an earlier relapse, patients with TP53 mutations could achieve a high CR rate to decitabine [19]. We then reported a high response (83.3%; 5/6 patients) to decitabine and 66 months of median RFS in NPM1^MUT^DNMT3A^WT^ patients [28]. In this assay, we again defined a special higher decitabine-induced CR in 14 BCOR^MUT^ patients (CR up to 57.1%; *p* = 0.036) and a much longer post-CR survival (40 months vs. 20 months; *p* = 0.036) when compared to those without BCOR mutations. Taken together, the therapeutic efficiency of decitabine would be optimized if we choose MDS patients with TP53 mutations, NPM1^MUT^ PDNMT3A^WT^, or BCOR mutations as first-line candidates for the decitabine treatment protocol. Notably, longer post-CR survival of the eight patients who achieve CR came from the premise that 3 patients who accepted no addition treatment after decitabine had an induced CR. We considered this reaction style to be very important and proposed a new theory about the medication method for hypomethylating agents. Perhaps a duly stop time but not a continuous application of HMAs, which can maintain an optimal epigenetic regulation state, will be the best choice for some special patients who achieved CR. From the finding in this assay, whether the special mutation locus (like P483L at exon 4) was resistant to decitabine, while coexistence of ASXL1 mutations resulted in a decitabine-induced response need to be examined in a study with a larger sample size to reach a conclusion.

## Conclusions

BCOR mutations seemed to play a role mainly in MDS patients with normal chromosomes, and biogenetically emerging after the initial but before the transformation related mutations. Which predict tAML transformation and poor prognosis when HMAs intervening is lack. Whereas BCOR^MUT^ MDS patients showed a sensitive response to the decitabine treatment protocol and achieved longer post-CR survival.

## Supplementary Information

**Additional file 1: Figure S1.** Dynamics of BCOR and concurrence gene mutations before and after AML transformation in six MDS patients. The VAF of BCOR mutation and other concurrence gene mutations are showed in red and green bar, respectively. The black bar shows the AML transformation-related gene mutaitons. **Table S1.** Characteristics of 43 patients with myelodysplastic syndromes harboring BCOR mutations.

**Additional file 2.** RNA sequencing data of bone marrow mononuclear cells from three MDS patients with BCOR mutation and three patients with BCOR wild type.

## Data Availability

The RNA sequencing dataset generated during the current study are available from the Additional file 2.

## Abbreviations

BCOR: BCL6 corepressor
MDS: Myelodysplastic syndrome
HMA: Hypomethylating agents
NC: Normal chromosome
RAEB: Refractory anemia with excess blasts
PRC: Polycomb repressive complex
OS: Overall survival
FAB: French–American–British
IPSS: International prognostic scoring system
CMML: Chronic myelomonocytic leukemia
IWG: International working group
NGS: Next generation sequencing
AML: Acute myeloid leukemia
VAF: Variant allele frequency
TFS: Transformation-free survival
HI: Hematological improvement
CR: Complete remission
