# Supplementary material for: Dynamics of epigenetic regulator gene BCOR mutation and response predictive value for hypomethylating agents in patients with myelodysplastic syndrome
Source: Clin Epigenetics. 2021 Aug 30;13:169. doi: 10.1186/s13148-021-01157-8 (PMC8404357; doi:10.1186/s13148-021-01157-8)
Supplement: Supplementary file 1 — Additional file 1: Figure S1. Dynamics of BCOR and concurrence gene mutations before and after AML transformation in six MDS patients. The VAF of BCOR mutation and other concurrence gene mutations are showed in red and green bar, respectively. The black bar shows the AML transformation-related gene mutaitons. Table S1. Characteristics of 43 patients with myelodysplastic syndromes harboring BCOR mutations. [file 13148_2021_1157_MOESM1_ESM.pdf]

Supplementary data 1

Table SⅠ Characteristics of 43 patients with myelodysplastic syndromes harboring BCOR mutations

Case No.	Gender	Age (year)	subtype	BM Blast (%)	Karyotype	BCOR mutations (VAF %)	Concomitant mutations
1	F	23	RAEB-2	18.0	Normal	BCOR/4/C925Y (51)	DHX9, WT1
2	M	80	RAEB-1	8.4	47,XY,+8[10]/46,XY[10]	BCOR/11/K1452X (60)	DNMT3A, ROBO1, STAG2
3	M	40	RAEB-2	3.6 (Auer)	46,XY,del(20)(q12q13)[20]	BCOR/8/R1323W (50)	NPM1
4	F	36	RAEB-1	5.0	Normal	BCOR/8/K1339N (33)	ASXL1, MPL, TET2, UPF3A
5	F	56	RAEB-1	8.0	Normal	BCOR/4/A619fs (16)	PHF6, RUNX1, TET2
6	M	77	RCMD	4.0	Normal	BCOR/4/Y361fs (85)	NRAS, ROBO1, RUNX1, U2AF1
7	M	56	RCMD	2.2	Normal	BCOR/12/R1547*(49)	ASXL1, RUNX1, SRSF2
8	M	62	RAEB-1	9.5	Normal	BCOR/4/p819-819del (74)	DNMT3A, IDH2, ROBO1, RUNX1
9	M	69	RAEB-2	11.2	Normal	BCOR/7/R1165X (29) /BCOR/11/C4326+1C>A (56)	CEBPA, DNMT3A, RUNX1, STAG2, TET2, U2AF1
10	M	57	RAEB-2	13.2	Normal	BCOR/7/P1101fs (65)	ITIH3
11	F	69	RAEB-2	17.0	Normal	BCOR/4/V912fs (32)	ASXL1, STAG2
12	F	43	RCMD	3.5	47,XX,add(3)(q28),+8[4]/46,XX[5]	BCOR/4/P483L (48)	EZH2, IDH1, U2AF1

13	F	73	RAEB-2	12.0	Normal	BCOR/4/T738fs (46)	EZH2, MPL, RUNX1
14	M	51	RAEB-1	8.2	46,XY,-11,+mar[10]/46,XY,del(7)(q31),inc[3]/46,XY[2]	BCOR/4/P483L (100)	No
15	F	20	RAEB-1	5.0	Normal	BCOR/10/P1367fs (30)	RBM, TET2,
16	F	44	MDS-U	0	Normal	BCOR/9/L1299fs (30)	CEBPA, TET2
17	M	21	RT	0.6	Normal	BCOR/8/L1299fs (30)	ASXL1, CALR, MPL
18	F	46	RAEB-2	15.0	Normal	BCOR/4/T591fs (45)	DNMT3A; IDH2,
19	M	70	RAEB-1	7.8	46,XY,inv(1)(q21q25),del(5)(q22q35[9]/46,XY[9]	BCOR/4/P483L (100)	DNMT3A, JAK2, RUNX1, TET2, TP53, U2AF1
20	M	68	RAEB-1	7.5	Normal	BCOR/4/T870fs (44)	ASXL1, DHX9, STAG2
21	F	52	RCMD	1.0	Normal	BCOR/7/R1165X (24)	No
22	M	46	RCMD	0.1	47,XY,+8[10]/46,XY[10]	BCOR/4/P483L (100)	ASXL1, JAK2, ANKRD11
23	M	18	RCMD	0.4	Normal	BCOR/8/G1342X (14)	IDH1
24	F	35	RCMD	0.5	Normal	BCOR/4/R810X (12)	ASXL1, IDH1
25	M	70	RCMD	1.8	Normal	BCOR/8/R1183X (39)	JAK2, STAG2
26	F	54	RCMD	0.4	Normal	BCOR/4/S526L (52)	EZH2, KIF20B, SETBP1
27	M	64	RT	2.0	46XY, +8[5]/46XY[15]	BCOR/15/K1672* (13)	ASXL1, STAG2, TET2
28	M	84	RAEB-1	6.0	Normal	BCOR/13/O1606X (21)	ASXL1, SRSF2
29	F	41	RCMD	3.0	Normal	BCOR/6/P1083fs (21.4)	No
30	F	54	RCMD	2.5	Normal	BCOR/4/P483L (46)	No
31	M	44	RT	2.5	Normal	BCOR/4/I501fs (60)	IDH2, U2AF1
32	F	77	RCMD	3.6	Normal	BCOR/4/P483L (80)	DNMT3A

33	M	61	RAEB-1	7.4	Normal	BCOR/10/1380C (62)	ASXL1
34	M	72	RCMD	1.0	Normal	BCOR/4/T819fs (91)	MPL, ROBO1
35	F	54	RAEB-2	19.0	44,XX,-2,-4,del(5)(q13q33),der(11),-16,-17,del(20)(q11),+2mar,inc[15]	BCOR/4/P483L (50)	SETBP1, TET2, TP53, ROBO2
36	F	65	RAEB-2	18.0	46,XX,del(20)(q12)[10]	BCOR/9/V1351M (54)	ASXL1, U2AF1
37	M	67	RAEB-1	5.0	Normal	BCOR/4/P483L (100)	ASXL1, U2AF1
38	M	77	RAEB-1	7.0	48,XY,+8,+2[2]/46,XY[18]	BCOR/13/Y1567fs (28)	ASXL1, TET2, RUNX1
39	M	69	RAEB-1	9.0	Normal	BCOR/4/G154fs (76)	CEBPA, DNMT3A, MPL, STAG2, WT1
40	M	69	RAS	0.6	Normal	BCOR/4/P483L (99.5)	DNMT3A, SF3B1
41	F	63	RT	0	47,XX+8[3]	BCOR/4/P483L (50)	No
42	M	73	RAEB-2	10.0	46,XY,t(9;21)(q22;q22)[8]/47,idem,+8[7]/46,XY[2]	BCOR/4/S637fs (40)	ASXL1, EZH2, DHX9, TET2
43	F	28	RCMD	2.6	47, XX, del (1)(p22), der(2), del(7)(q31), +8, der(17)[8]/46,XX[10]	BCOR/4/R861H (49)	No

Figure S1

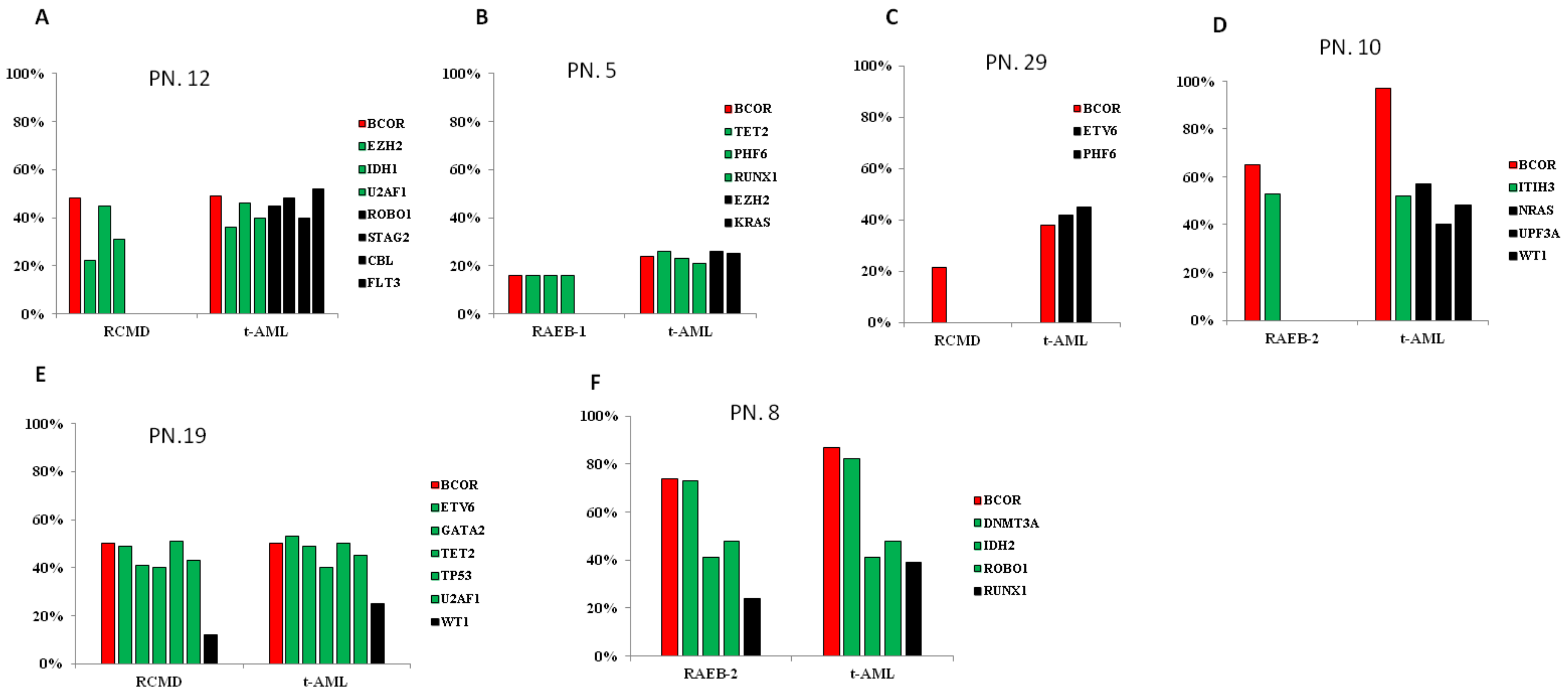
A
PN. 12
100%
80%
60%
40%
20%
0%
RCMD
t-AML
BCOR
EZH2
IDH1
U2AF1
ROBO1
STAG2
CBL
FLT3
B
PN. 5
100%
80%
60%
40%
20%
0%
RAEB-1
t-AML
BCOR
TET2
PHF6
RUNX1
EZH2
KRAS
C
PN. 29
100%
80%
60%
40%
20%
0%
RCMD
t-AML
BCOR
ETV6
PHF6
D
PN. 10
100%
80%
60%
40%
20%
0%
RAEB-2
t-AML
BCOR
ITIH3
NRAS
UPF3A
WT1
E
PN.19
100%
80%
60%
40%
20%
0%
RCMD
t-AML
BCOR
ETV6
GATA2
TET2
TP53
U2AF1
WT1
F
PN. 8
100%
80%
60%
40%
20%
0%
RAEB-2
t-AML
BCOR
DNMT3A
IDH2
ROBO1
RUNX1
